# CollecTF: a database of experimentally validated transcription factor-binding sites in Bacteria

**DOI:** 10.1093/nar/gkt1123

**Published:** 2013-11-14

**Authors:** Sefa Kılıç, Elliot R. White, Dinara M. Sagitova, Joseph P. Cornish, Ivan Erill

**Affiliations:** Department of Biological Sciences, University of Maryland Baltimore County (UMBC), 1000 Hilltop Circle, Baltimore, MD 21250, USA

## Abstract

The influx of high-throughput data and the need for complex models to describe the interaction of prokaryotic transcription factors (TF) with their target sites pose new challenges for TF-binding site databases. CollecTF (http://collectf.umbc.edu) compiles data on experimentally validated, naturally occurring TF-binding sites across the Bacteria domain, placing a strong emphasis on the transparency of the curation process, the quality and availability of the stored data and fully customizable access to its records. CollecTF integrates multiple sources of data automatically and openly, allowing users to dynamically redefine binding motifs and their experimental support base. Data quality and currency are fostered in CollecTF by adopting a sustainable model that encourages direct author submissions in combination with in-house validation and curation of published literature. CollecTF entries are periodically submitted to NCBI for integration into RefSeq complete genome records as link-out features, maximizing the visibility of the data and enriching the annotation of RefSeq files with regulatory information. Seeking to facilitate comparative genomics and machine-learning analyses of regulatory interactions, in its initial release CollecTF provides domain-wide coverage of two TF families (LexA and Fur), as well as extensive representation for a clinically important bacterial family, the *Vibrionaceae*.

## INTRODUCTION

The binding of transcription factors (TF) to target sites in genomic DNA is a defining element of transcriptional regulatory networks (TRN). Bacterial transcription networks rely extensively on direct TF–DNA interactions and display relatively lower complexity than their eukaryotic counterparts (1,2). This has generated substantial interest in modeling and cataloguing bacterial TF-binding site interactions, leading to the emergence of several model organism-based databases aimed at compiling TF-binding sites to define TRN and explore their interplay with other cellular systems (3–6), as well as two databases dedicated to compiling gene-regulation information across multiple prokaryotic species (7,8). In recent years, the development of high-throughput techniques for the identification of TF-binding sites, like ChIP-Seq (9), has led to a rising influx of data that challenges the traditional manual curation process and the underlying definition of TF-binding sites in current databases (3). In turn, the increasing availability of such data and the mounting evidence on the complexity of TF-binding site recognition and regulatory logic in bacteria (10–12) have led to the proliferation of machine learning and comparative genomics techniques to model TF-binding site interactions (13–15). These approaches require the integration of genome-wide, multi-species knowledge on TF binding, using a broader definition of TF-binding site and having direct access to the experimental sources of evidence for each site. At present, cross-species information is hard to compile and standardize across prokaryotic TF-binding site databases. Different databases use different ranking systems for experimental evidence, which is not always made directly accessible, and will often combine experimentally supported sites with computational predictions, as well as naturally occurring sites with artificially generated ones. CollecTF seeks to address these issues and complement existing databases by providing high-quality, transparent annotation on naturally occurring TF-binding sites across the Bacteria domain, explicitly integrating multiple sources of evidence and binding site definitions into a highly accessible, machine readable and fully customizable database. In line with previous initiatives (16), CollecTF combines in-house curation with direct submission from authors, and seeks to foster a sustainable external submission model through its integration with NCBI RefSeq (17).

## DATABASE CURATION

**Figure 1.**
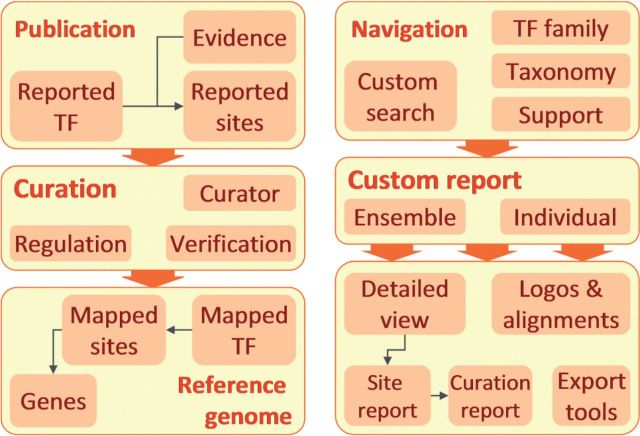
Schematic representation illustrating the CollecTF data structure, curation and navigation processes. (Left panel) The curation table is the pivotal element of the relational design in CollecTF, providing a central link to all the other tables in the database (Supplementary Figure S1) and establishing a link between reported TF-binding sites, the evidence supporting them, their regulatory effects on genes and their mapped instances in a reference genome. (Right panel) Navigation is initiated by browsing or customized search, leading to a dynamically generated report that can be cumulative or individualized for each TF/species pair (Supplementary Figure S2). Motif alignments and logos are provided for visualization, together with export functions to FASTA and flat-file formats. Users can link out to specific site reports and link-out to curation reports to evaluate all the supporting evidence for reported sites and the genome mapping process.

The main goal of CollecTF is to provide high-quality annotation on experimentally validated TF-binding sites in their genomic context. This is accomplished through the manual curation of peer-reviewed literature with a special focus on the experimental process used to identify TF-binding sites. Curations may be initiated in-house by a team of curators specifically trained on the experimental techniques used to determine TF-binding sites, or by direct submission from authors. In all cases, curations are double-checked by at least two curators following a well-defined set of guidelines before inclusion in the database. Given its fundamental importance, the curation process is the central element of the relational database structure of CollecTF (Figure 1 and Supplementary Figure S1). A curation essentially establishes a link from a TF and a number of sites as reported in a scientific publication to a set of genome positions and a protein accession in the NCBI RefSeq database. The mapping process involves an automated search of reported sites in the target reference sequence and manual verification of their genomic location by the curator. Exact matching sequences for each binding site are first identified in the reference genome and their genomic location is reported to the curator together with information on the functional annotation of nearby genes. The curator uses this contextual information to validate the correspondence between identified genome positions and reported sites. For reported sites without exact matches in the genome, a search allowing up to two mismatches is performed. Identified sequences are again annotated with contextual information, ranked using a position-specific weight matrix derived from the list of reported sites and presented to the curator for manual verification. For any given curation, at least 90% of the reported sites must have exact matches in the reference genome in order for the mapping to be accepted. Otherwise, the submitted data is stored but not associated with the NCBI record. Most importantly, for each TF-site pair the curation process also determines all the techniques used to identify the site and its regulatory role on nearby genes, and generates a summary description of the experimental process. CollecTF distinguishes between two primary experimental sources: direct evidence of binding and evidence of TF-mediated regulation. *In silico* techniques are annotated as complementary sources in the experimental process, but are not admitted as the single source of evidence for a record. CollecTF does not rank experimental techniques. Instead, it provides direct queryable access to the experimental support, providing users with full control over quality standards of retrieved data.

## DATABASE CONTENT

CollecTF contains exclusively experimentally verified TF-binding sites identified in natural DNA sequences. Historically, the definition for TF-binding site has been associated to the presence of a well-defined TF-binding motif, but in the last decade it has become increasingly apparent that many TFs bind DNA without clear-cut sequence determinants (10–12). To accommodate this diversity without losing the high-quality annotation of motif determinants, CollecTF curators classify sites as motif associated or non-motif associated. Motif associated sites are either explicitly identified by authors as following a pre-established motif or shown by means of detailed experimental work (e.g. site-directed mutagenesis) to conform to a new motif. Non-motif associated sites are any DNA fragments conclusively identified by the authors as bound to the TF; this includes ChIP sites as reported by authors using a given peak-calling method and confidence interval. Any quantitative information associated with sites (e.g. estimated *K_d_*) is also stored during curation, together with its range and a short description of the quantitative technique. For ChIP data, additional information on the experimental conditions and the ChIP protocol is also compiled.

Experimental evidence of binding for a given genomic position may be distributed across multiple reported sites of either type. Wherever a motif associated site has been defined, CollecTF dynamically combines multiple sources of evidence by arbitrarily defining a leader site and using two simple pair-wise propagation rules. Evidence from two motif associated sites is combined if the overlap between sites is >75% of the combined site length. Evidence from non-motif associated sites is integrated into a leader site if they fully overlap any of the combined motif associated sites. CollecTF was born with the goal of unifying and simplifying data collection on TF-binding sites for comparative genomics and machine learning approaches. In its initial release, CollecTF curators have focused on providing comprehensive, domain-wide coverage for two TF families (LexA and Fur), as well as extensive TF representation for a bacterial family, the *Vibrionaceae*, for which abundant knowledge on the intertwined transcriptional regulation of virulence is available in the literature but poorly represented in available databases (18). CollecTF plans to steadily increase its coverage by targeting other bacterial groups of clinical and agricultural relevance not covered by organism-based databases, such as the *Xanthomonadaceae* or the Campylobacterales, focusing on TF families with substantial experimental support across multiple species, such as OmpR and LuxR, and promoting direct author submissions on any transcriptional system/species. At the time of writing, CollecTF contains >2000 curated sites for 68 TFs in 64 species.

## NAVIGATION AND AVAILABILITY

**Figure 2.**
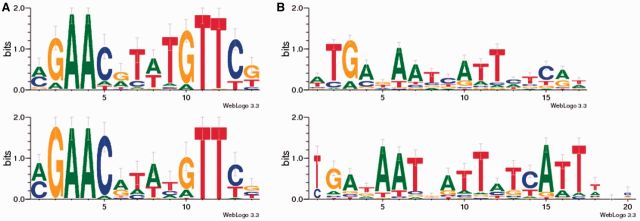
(**A**) Sequence logo for LexA-binding sites in the Firmicutes (top) and in *Bacillus subtilis* (bottom). (**B**) Sequence logo for Fur-binding sites with experimental evidence of binding (top) or experimental evidence of regulation (bottom). Both examples are extracted from dynamically generated CollecTF reports and illustrate the ability to customize queries and the fluidity inherent to the concept of TF-binding motif in CollecTF.

**Figure 3.**
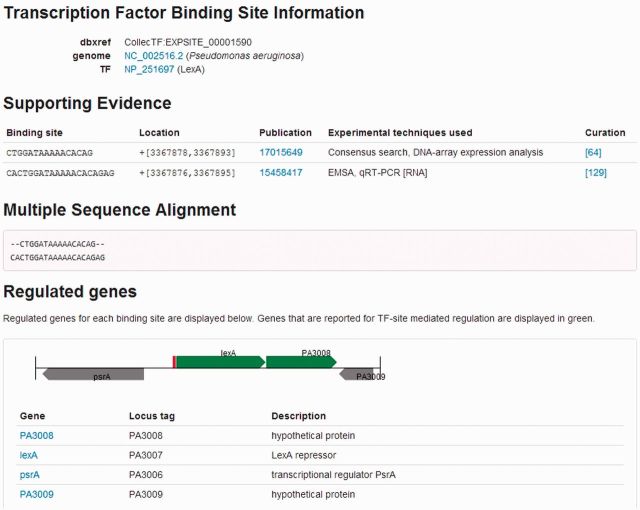
Snapshot of the site report page for a *Pseudomonas aeruginosa* LexA-binding site, illustrating the integration of supporting experimental evidence and including out-links to curations, publications, technique descriptions and NCBI Gene records. Like all other site report pages, this record is directly accessible through its *db_xref* link at http://collectf.umbc.edu/EXPSITE_00001590.

CollecTF (http://collectf.umbc.edu) is designed to maximize ease of access to TF-binding site data in both human- and machine-readable formats. Users can browse the database taxonomically, by TF family or by experimental techniques, or search clades and TF families for sites with specific types of experimental support (e.g. all Fur sites in *Pseudomonas* identified through mobility shift assays). Users can elicit reports at any time during browsing or searching, and have the option of condensing the report or reporting by individual species/TFs. Instead of relying on pre-computed motif representations, motif associated sites are realigned dynamically with LASAGNA and displayed with WebLogo (19,20), providing a fluid representation of TF-binding motifs that incorporates all the available sources of evidence selected by the user (Figure 2). All report pages offer a detailed view of TF-binding sites in their genomic context with out-links to site description and gene NCBI accessions, as well as export options to FASTA, flat-file CSV and ARFF sequence formats and multiple position-specific matrix formats (Supplementary Figure S2). CollecTF also features a suite of motif comparison tools that allow users to assess the similarity of motifs derived from independent queries. Site-based comparisons contrast the distribution of pair-wise Levenshtein distances within and between motifs (21). Motif-based comparisons combine ungapped Smith–Waterman motif alignment with several well-established statistics, such as the average Kullback–Leibler divergence or the average log-likelihood ratio, which are contextualized by permutation tests on the aligned motifs (22). All data stored in CollecTF is directly accessible through navigation. Navigating from main report pages, users may inspect the individual site instances contributing support for a specific leader site in a motif alignment or access the curation record including curation notes, out-links to external databases for supporting evidence and both the originally reported and the genome-mapped data (Figure 3). Flat-file versions of CollecTF primary tables are generated periodically and made freely available for download.

### Integration with NCBI RefSeq

**Figure 4.**
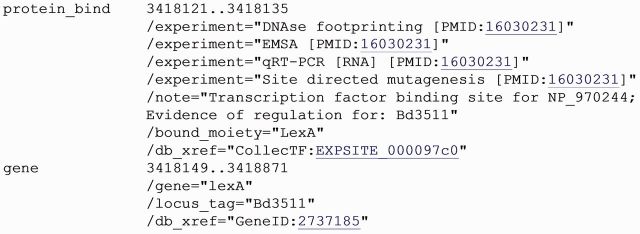
Detail of the CollecTF generated record for the *Bdellovibrio bacteriovorus* complete genome (RefSeq accession NC_005363.1) showing the */bound_moiety* feature corresponding to a LexA-binding site upstream of the *lexA* gene (Bd3511). The feature details the experimental evidence for the site with associated PubMed identifiers and provides a *db_xref* out-link to CollecTF.

Bioinformatics databases suffer from intertwined visibility and sustainability problems that limit their half-life and up-to-dateness, and can lead to the so-called data tomb effect (23). CollecTF seeks to partially address these problems by incorporating periodic releases into the NCBI RefSeq database as part of an ongoing collaboration. TF-binding sites with experimental evidence of binding are incorporated into complete genome RefSeq records as */bound_moiety* features detailing the experimental technique supporting each site and encoding a *db_xref* out-link to their CollecTF record (Figure 4). The integration of CollecTF with NCBI RefSeq addresses the historical absence of this type of information in the largest and most frequently used bioinformatics resource globally. At the same time, it dramatically increases the visibility of the CollecTF database and its content. Embedding data on regulatory interactions directly in complete genome records allows reaching a much wider audience that is often unaware of more specialized databases. As such, it has the potential to provide unanticipated insights and trigger connections and discoveries in molecular microbiology research. Increasing the visibility of the database and making it available on genome records with direct references to the original publications also provides an incentive for authors to submit their contributions to CollecTF. By encouraging direct submissions through increased visibility and concerted dissemination efforts, CollecTF seeks to alleviate the pressure on in-house curations and to develop a sustainable model that allows database growth and expansion while maintaining up-to-date information on TF-binding sites.

## SUPPLEMENTARY DATA

Supplementary Data are available at NAR Online.
